# Neural Mechanisms of Object Location Memory in Huntington's Disease

**DOI:** 10.1002/mds.30232

**Published:** 2025-05-28

**Authors:** Yifat Glikmann‐Johnston, Garance Delagneau, Tamrin Barta, Julie C. Stout, Adeel Razi

**Affiliations:** ^1^ Turner Institute for Brain and Mental Health and School of Psychological Sciences, Monash University Clayton Australia; ^2^ Wellcome Centre for Human Neuroimaging, University College London London United Kingdom; ^3^ CIFAR Azrieli Global Scholars Program, CIFAR Toronto Ontario Canada

**Keywords:** caudate nucleusfunctional MRIputamenspatial memorytemporal lobe|

## Abstract

**Background:**

Object‐location memory impairment in Huntington's disease (HD) occurs from premanifest period and declines as HD progresses, however, pathogenesis of object‐location memory is unknown. The striatum and hippocampus are affected in HD, functionally interacting allowing intact object‐location memory.

**Objectives:**

The present study investigated if object location memory impairment in premanifest and manifest HD was associated with aberrations in effective connectivity between striatum and hippocampal formation.

**Methods:**

Using dynamic causal modelling, we examined effective connectivity between the striatum and hippocampus and association with object‐location memory in 35 HD participants (23 premanifest, 12 early manifest) and 32 controls.

**Results:**

HD participants' object‐location memory was worse than controls, with performance associated with aberrant effective connectivity from the striatum to hippocampal formation, lower connectivity was associated with poorer object‐location memory. Connectivity from hippocampal formation to the striatum was lower in manifest HD. In premanifest HD, connectivity from parahippocampal gyrus to the striatum was stronger and associated with better object‐location memory.

**Conclusions:**

Findings raise questions regarding compensatory neural processes in HD and other neurodegenerative diseases, providing pathophysiological evidence that cognitive impairment may be related to connectopathy. © 2025 The Author(s). *Movement Disorders* published by Wiley Periodicals LLC on behalf of International Parkinson and Movement Disorder Society.

Spatial memory impairment occurs in Huntington's disease (HD) before the emergence of motor signs in premanifest HD and becomes more pronounced as HD progresses into manifest stages where motor signs are apparent.^1^ The impact of spatial memory impairment on daily life is significant. “For me, it started with small, unexplained absences: car keys, glasses, a million lighters, shoes, clothes. Then I lost the world, city by city. Familiar places became a scary tangle of streets, so I stayed in the house.” (Extracted from The Guardian by Charlotte Raven, Living with Huntington's disease: “For our family, the end of days is always close at hand”, October 16, 2021). Remembering where things are in the environment is called object location memory, which is a component of spatial memory essential for many life activities. We have recently shown that object location memory impairment in manifest HD and in premanifest HD many years from estimated motor onset (>10 years).^1^ This finding is important because 10 to 20 years before motor onset, people are thought to be asymptomatic with intact cognition.^2^ Having detected changes in object location memory in presymptomatic HD may suggest this cognitive domain is highly sensitive to HD, however, the pathogenesis of object location memory in HD is unknown.

Neurocognitively, the striatum (caudate nucleus, putamen, and ventral striatum) and hippocampal formation (hippocampus proper and adjacent parahippocampal cortices) are key brain structures for both object location memory and HD. Atrophy in the striatum is the hallmark radiological finding in HD.^3^ The hippocampus also undergoes early atrophy at a smaller magnitude compared to the striatum, and mostly after motor onset.^4^, ^5^ Whole brain diffusion magnetic resonance imaging (MRI) did not show connectivity loss between the hippocampus and striatum in either premanifest or manifest HD^6^ suggesting that the functional disconnection between the striatum and hippocampus may occur before hippocampal structural loss. Both hippocampus and striatum mediate spatial memory, including object location memory.^7^ The striatum implements object location memory in relation to one's body location and objects in the environment, termed egocentric spatial mapping.^7^ The hippocampal formation implements spatial mapping through a cognitive map of objects within the environment, independent of one's body orientation, termed allocentric.^8^ The striatum and hippocampus are connected anatomically and interact functionally to enable intact spatial memory, including object location.^9^

In HD, the interaction between the striatum and hippocampus during spatial memory has been shown in one publication, which demonstrated altered connectivity during route recognition, a component of spatial memory.^10^ Voermans et al^10^ showed premanifest and manifest HD participants video sequences of first‐person fixed virtual home routes followed by route recall. HD participants maintained close to normal performance, however, underlying brain activity varied between groups. Compared to controls, task‐based functional MRI (fMRI) showed that HD participants had higher hippocampal activity, alongside reduced functional connectivity between the caudate nucleus and hippocampus. The authors suggested a compensatory process helps to overcome the declining function of the caudate nucleus to maintain normal route recall. Although these results highlight the cooperation between the striatum and hippocampus in spatial memory, they do not establish the direction or valence (eg, inhibition or excitation) of the interaction. Further, given that spatial memory is not a unitary or internally homogenous and includes many different components such as navigation, object location, and route recognition, we cannot assume the observed striatum‐hippocampus cooperation in route recognition will be seen in other spatial memory components or in the domain as a whole.^11^

Directional and functional interactions between the striatum and hippocampus, and their association with object location memory, can be studied using effective connectivity. Effective connectivity refers to the causal influences one neural system applies over another,^12^, ^13^ and can be estimated using dynamic causal modelling (DCM). DCM uses a Bayesian framework for developing a generative model of directed or causal influences between brain regions comprising spatially distributed neural systems.^12^ Here, we used spectral DCM^13^, ^14^, ^15^ to characterize striatum‐hippocampal formation effective connectivity in premanifest HD, early manifest HD, and matched controls using resting‐state fMRI data. DCM has been successfully used to demonstrate effective connectivity alterations in HD, including fMRI motor task‐induced connectivity changes predictive of disease progression.^16^ Based on previous HD DCM study with a cognitive task showing decreased connectivity in a network underlying verbal working memory,^17^ we tested two hypotheses: (1) there would be reductions in connectivity between the striatum and hippocampal formation in premanifest and manifest HD; and (2) reduced striatum‐hippocampal formation effective connectivity would be associated with worse object location memory in HD.

## Subjects and Methods

### Participants

This study included 35 HD gene expansion carriers, including 23 premanifest HD and 12 early manifest HD, and a comparison group of 32 healthy controls, using the sample from Glikmann‐Johnston et al,^11^ with available fMRI and object location memory data. There were more premanifest HD participants who could fulfil and study inclusions and perform fMRI and cognitive testing than manifest HD participants, hence the unequal sample size of the HD groups. HD participants, age 30 to 66 years with genetically confirmed CAG expansions (≥36 repeats), were recruited through Calvary Health Care Bethlehem Hospital and ENRU‐Stout HD data base at Monash University (Melbourne, Australia). Exclusion criteria included major neurological illness (except HD), significant head injury, or non‐HD‐related psychiatric disturbances. Premanifest HD participants had never received clinical diagnosis of HD, as indicated by the Unified Huntington's Disease Rating Scale (UHDRS) diagnostic confidence levels <4.^18^ Early stage HD participants showed minimal to moderate clinical impairments as indicated by the UHDRS total functional capacity (TFC) scores of >7.^18^

None of the premanifest HD and 21.4% of our early stage HD participants were taking dopamine antagonists, and 12.5% of premanifest HD and 42.8% of early stage HD participants were taking antidepressant medication at the time of study participation. Because of the different types and varying doses of medication in these groups, it was not possible to add these as covariates in the analysis. Nevertheless, we examined the association between medication and object location memory, our main focus here, and directly compared those taking to those not taking either antidepressants or dopamine antagonists. This analysis produced non‐significant result (*P* > 0.1), therefore, we concluded that medication did not significantly influence performance in this study.

Given the progressive nature of HD, the manifest HD group was older than the premanifest HD group (*t*(33) = −3.28, *P* = 0.002), had higher UHDRS total motor scores (TMS) (*t*(10.69) = −6.1, *P* < 0.001), lower UHDRS TFC scores (*t*(11.66) = 2.51, *P* = 0.028), and higher disease burden scores (DBS) (given by: age × CAG‐35.5;^19^ *t*(33) = − 2.39, *P* = 0.022) than the premanifest HD group. Gender distribution between premanifest and manifest HD groups did not differ significantly (χ^2^(1, n = *35*) = 2.394, *P* = 0.122). Control participants were matched to individual HD participants based on age (within 5 years), handedness, gender, and estimated intelligence quotient (IQ) (within the same range). At group level analyses (controls vs. HD group combined of premanifest and manifest) there were no differences in age (*t*(65) = −0.20, *P* = 0.846), gender (χ^2^(1, n = 67) = 0.014, *P* = 0.907), handedness (χ^2^(*1*, n = 67) = 0.130, *P* = 0.718), or estimated IQ (*t*(65) = 1.38, *P* = 0.171). The groups did not differ in depressive symptomology (*t*(65) = −1.52, *P* = 0.879) (Table 1). All participants gave written informed consent in accordance with the Declaration of Helsinki. Procedures were approved by the Human Research Ethics Committees of Monash University and Calvary Health Care Bethlehem Hospital, Melbourne Australia.

**TABLE 1:** Participant characteristics and clinical data

		Premanifest HD (n = 23)	Manifest HD (n = 12)	Controls (n = 32)
Age	Mean (SD)	45.61 (8.25)	55.17 (7.99)	48.47 (8.06)
	Median	46	55	49.50
	Range	30–59	43–66	35–65
Female	%	60.9	33.3	50
Right‐handed	%	87.5	100	93.80
Estimated FSIQ^a^	Mean (SD)	106.65 (9.39)	109.08 (9.15)	110.50 (8.52)
	Median	107	108.5	111.50
	Range	87–126	95–123	91–127
CESD‐R scale	Mean (SD)	8.96 (11.92)	9 (9.70)	8.59 (8.98)
	Median	4	6	5.50
	Range	0–42	0–29	0–37
CAG repeats	Mean (SD)	41.78 (1.81)	41.92 (1.44)	N/A
	Median	42	42	N/A
	Range	38–45	39–44	N/A
DBS	Mean (SD)	280.76 (83.27)	346.67 (63.70)	N/A
	Median	260	337.75	N/A
	Range	140–420	227.5–484.5	N/A
UHDRS TMS confidence	Mean (SD)	0.14 (0.36)	3.91 (0.30)	N/A
	Median	0	4	N/A
	Range	0–1	3–4^b^	N/A
UHDRS TMS frequency	Mean (SD)	1.33 (2.43)	19.18 (9.54)	N/A
	Median	0	19	N/A
	Range	0–8	9–40	N/A
UHDRS TFC	Mean (SD)	12.74 (0.62)	10.83 (2.59)	N/A
	Median	13	12.50	N/A
	Range	11–13	7–13	N/A

Abbreviations: HD, Huntington's disease; FSIQ, Full Scale Intelligence Quotient; CESD‐R, Center for Epidemiologic Studies Depression Scale‐Revised;^19^ CAG, cytosine adenine guanine; DBS, disease burden score;^17^ UHDRS, Unified Huntington's Disease Rating Scale; TMS, total motor score;^16^ TFC, total functional capacity.

^a^ FSIQ was estimated using the National Adult Reading Test second edition.^18^

^b^ One participant obtained a score of 3 (= motor abnormalities that are likely signs of HD with 90%–98% confidence) because of a leg injury that happened close to the motor assessment, which made it difficult to establish 99% confidence (score of 4).

### Procedure

Testing took 2 hours and was consistent for all participants. The first hour included testing of object location memory via a virtual house.^1^ Then, participants underwent a 30‐minute MRI protocol. During scanning, participants lay still and could close their eyes for the structural scans. Next, participants completed clinical examination, including an assessment of premorbid IQ using the National Adult Reading Test second edition,^20^ screening for depression with the Center for Epidemiologic Studies Depression Scale‐Revised,^21^ and other cognitive testing (including Rey Complex Figure Test, Paired Associates Learning, Symbol Digits Modalities Test, see Glikmann‐Johnstons et al^11^). A trained cognitive examiner administered clinical assessments. To minimize bias, participants were assigned a number, and analyses were conducted using unique participant identifications (IDs).

### Resting‐State fMRI

#### Brain MRI Acquisition and Pre‐Processing

MRI scanning was conducted using a 3‐Tesla Siemens Skyra MRI scanner (Siemens Magnetom, Malvern, PA, USA) at Monash Biomedical Imaging Research Centre (Melbourne Australia). Images acquired included T1‐weighted images (echo spacing = 6.3 ms, repetition time [TR] = 2300 ms, echo time [TE] = 2.07 ms, flip angle = 9°, slice thickness = 1.0 mm, interpolated voxel size = 1.0 × 1.0 × 1.0 mm^3^, field of view [FOV] = 256 mm, acquisition time = 7 minutes, 50 seconds) and resting‐state functional data (BOLD echoplanar imaging, 42 slices, TR = 2350 ms, echo spacing = 0.65 ms, TE = 30.0 ms, flip angle = 90°, slice thickness = 3.0 mm, interpolated voxel size = 3.0 × 3.0 × 3.0 mm^3^, FOV = 192 mm, acquisition time = 8 minutes, 1 second). During resting‐state fMRI, examiners instructed participants to follow an eyes‐open resting‐state protocol while fixating a black cross.

A total of 201 functional images were collected. The first five echo planar imaging sequences were discarded to allow for steady‐state equilibrium, leaving 196 volumes. Resting‐state fMRI were analyzed using statistical parametric mapping (SPM12) (https://www.fil.ion.ucl.ac.uk/spm/) in MATLAB 2018b (The MathWorks, Natick, MA, USA). The preprocessing pipeline included slice‐time correction (using the central slice of each volume as a reference), realignment to the first functional volume of each session, spatial normalization to Montreal Neurological Institute (MNI) space, and spatial smoothing by a 6 mm full‐width half‐maximum Gaussian kernel. Head motion was considered acceptable for all participants (<3 mm).

#### Selection and Extraction of Volumes of Interest

Eight brain regions were identified as implicated in spatial memory and HD: bilateral hippocampi, parahippocampi, caudate nuclei and putamen.^7^, ^22^, ^23^, ^24^, ^25^ Masks were created for each region of interest (ROI; left/right separately). Parahippocampus masks were created using WFU PickAtlas.^26^ Caudate nuclei, putamen, and hippocampi were manually segmented using ITK‐SNAP.^27^ This procedure has been described in detail elsewhere.^11^ In brief, segmentation was based on a standard atlas^28^ and followed validated protocols previously described (caudate nucleus,^29^ putamen,^30^ and hippocampus^31^). Using ITK‐SNAP, a total volume (mm^3^) was obtained for each brain structure. Volumes were normalized per Free et al^32^ to account for individual differences in head size (for each hemisphere separately). Analyses were performed using the normalized volumes. Researchers performing manual segmentation were blinded to group allocation. Intra‐class correlation coefficient values, used to measure inter‐rater reliability, ranged between 0.97 (left caudate nucleus) and 0.99 (left putamen).

### Spectral DCM

#### First Level Spectral DCM Analyses

Effective connectivity was estimated using spectral dynamic causal modelling (spDCM) for resting‐state fMRI, which was specified and inverted using DCM12 (Supplementary Data S1). We modelled data using a Bayesian hierarchical random‐effects model. At the first level, a fully connected model (64 connections) was created for each participant. We estimated the DCMs using spDCM, fitting the cross‐spectral density using a parameterized power‐law model of endogenous neural fluctuations. This analysis provided measures of directed (causal) interactions between regions.^14^

#### Second Level spDCM Analyses

Once the individual level spDCM were computed, group‐level analysis was performed using a parametric empirical Bayes (PEB) framework to characterize how group differences in neural circuitry were modulated by HD classification.^33^ Three groups were compared: controls, pre‐manifest HD, and manifest HD. Bayesian model reduction was used to test all combinations of parameters within each parent PEB model and “pruned” redundant model parameters.^33^ A Bayesian model averaging (BMA) approach quantified the strength of model connectivity parameters among regions.^33^ Only parameters exceeding the threshold of posterior probability >0.95, corresponding to strong evidence, were interpreted. Gender was used as a covariate in all group‐level analyses.

Leave‐one‐out cross validation was performed for each connection that reached the significance criterion—posterior probability >95—from the group level analyses.^34^ Analyses were performed separately for each group, and fitted the PEB model in all but one participant to predict variables of interest for the left‐out participant. This is repeated with each participant to assess averaged prediction accuracy.

### Object Location Memory

**FIG. 1:**
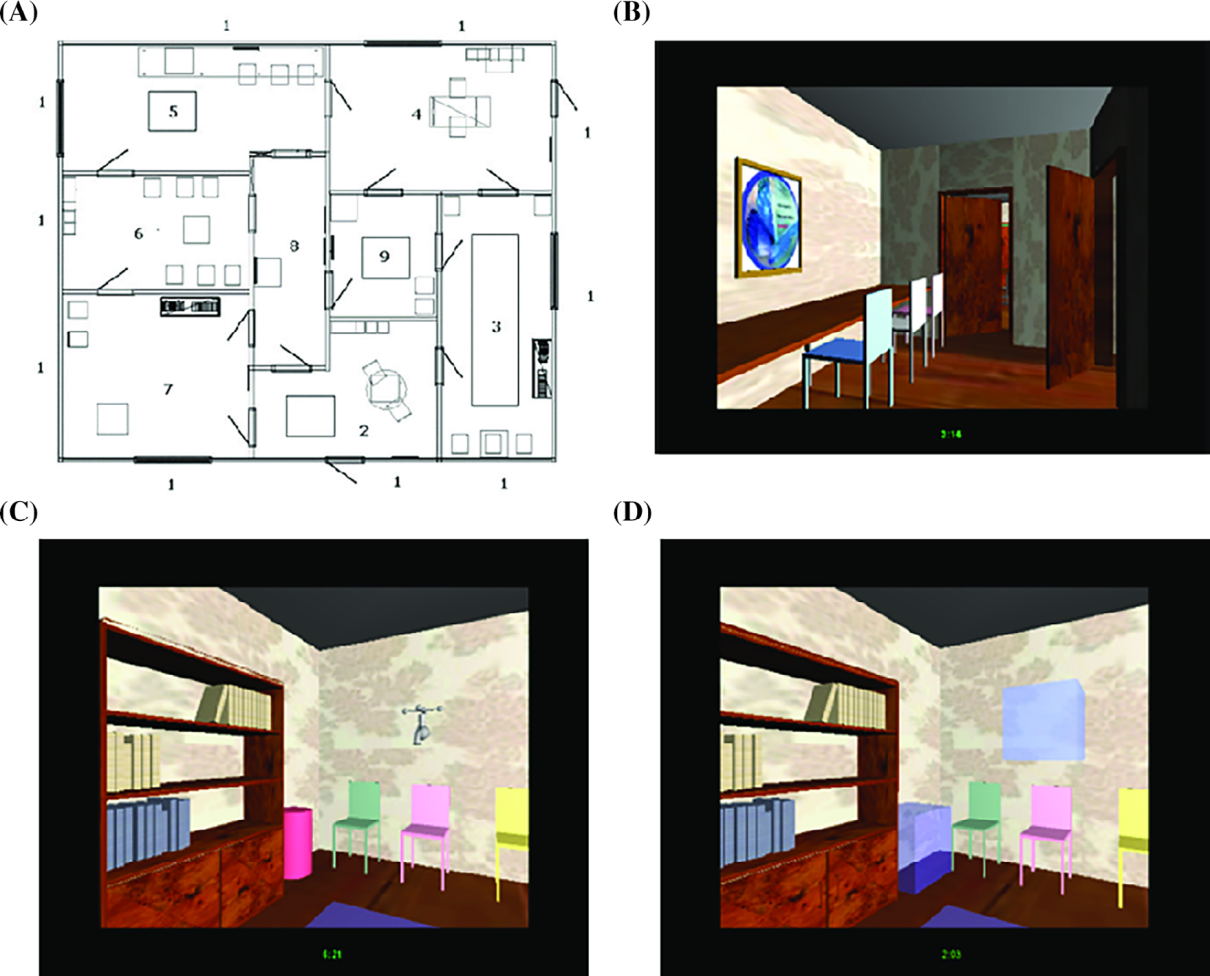
The virtual house. (**A**) Floor plan; (**B**) a view of conventional items located within the house (eg, chairs, table, and painting); (**C**) a room where test objects are present (tap, pink cylinder); and (**D**) where test objects are absent in the object location memory task. [Color figure can be viewed at wileyonlinelibrary.com]

Object location memory was tested using the virtual house, which mimics spatial demands of everyday life.^1^, ^11^ The house was a square structure comprising eight spaces (Fig. 1). Each space contained objects in conventional positions (eg, a picture on the wall, a chair at a table) and additional objects in arbitrary locations (eg, a big tap hanging off the wall, a model ship on a table). These objects formed test objects within the task. Of 11 test objects, three were geometric shapes (yellow sphere, pink cylinder, and blue rectangle), and eight were common objects (ship, tap, model car, shark, flower vase, balloon, piano, and fire extinguisher). The three shapes appeared 15 times in various locations, and each common object appeared once and in one room. Views to the environment exterior of the house varied according to the cardinal compass points toward which they were oriented.

The virtual house task began with a participant‐directed exploration phase, lasting 30 minutes minimum and 45 minutes maximum. An examiner was present while participants explored the virtual house. We did not observe effects of motor deficits in our HD cohort on operating the joystick, although latency effects were not measured. Testing object location memory immediately followed as well as other spatial components.^1^ Participants viewed a screen displaying the test objects, which the examiner named aloud. Participants then re‐entered the house and were taken on a prescribed route by the examiner (who controlled the joystick), where test objects had been removed and replaced by a pale blue three‐dimensional transparent box (Fig. 1D). All conventionally placed objects (tables, chairs, and pictures) remained in the virtual house. Participants were instructed to recall missing test objects that had occupied the position marked by a pale blue transparent box. The outcome measure was recall of each object in its correct location, which had a maximum score of 23 points.

To compare object location memory performance between groups on the virtual house we used one‐way analysis of covariance (ANCOVA) with post hoc pairwise comparisons for main effect and Bonferroni adjustment for multiple comparisons.

## Results

### Group Differences in Striatal‐Hippocampal Formation Effective Connectivity

**FIG. 2:**
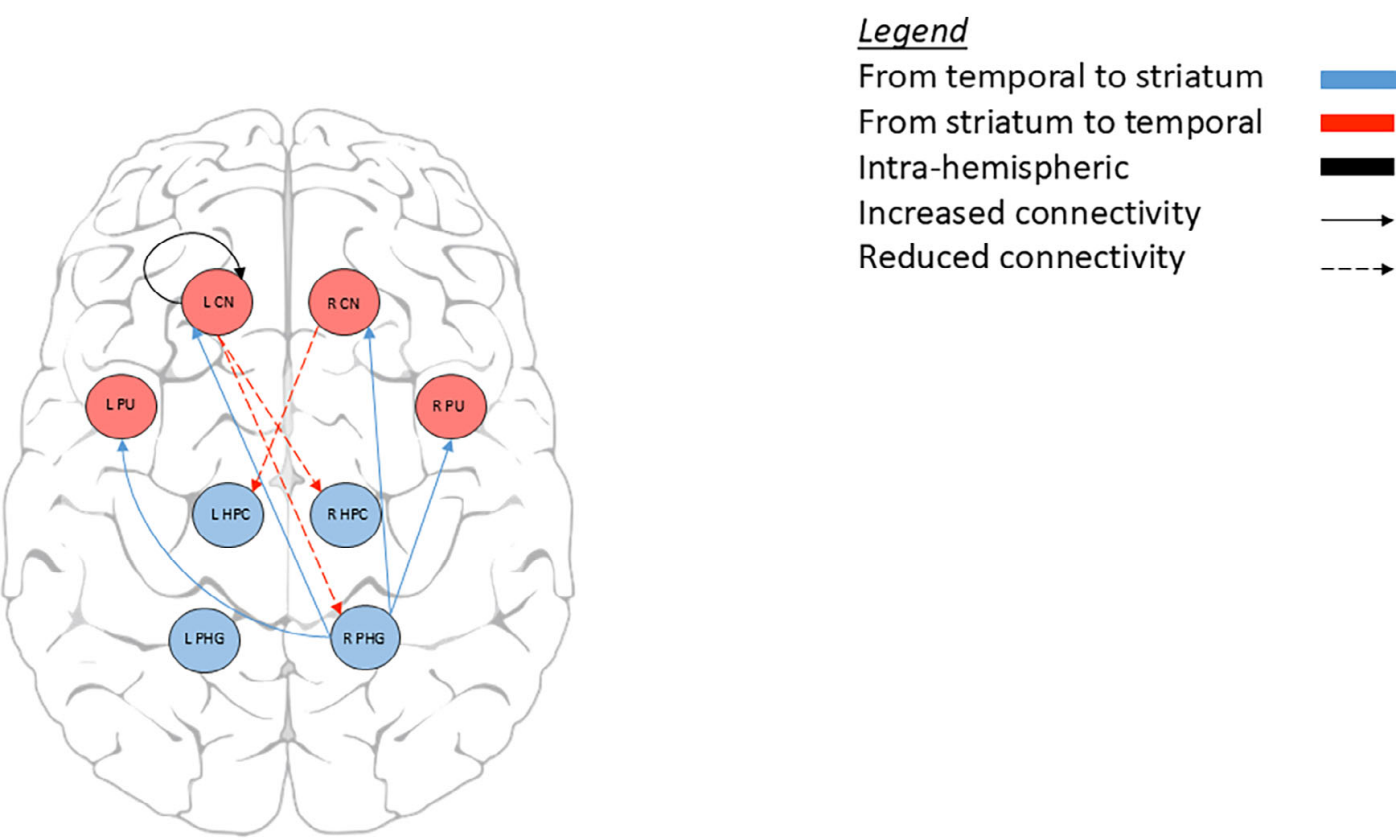
Effective connectivity in premanifest Huntington's disease participants compared to controls. [Color figure can be viewed at wileyonlinelibrary.com]

**FIG. 3:**
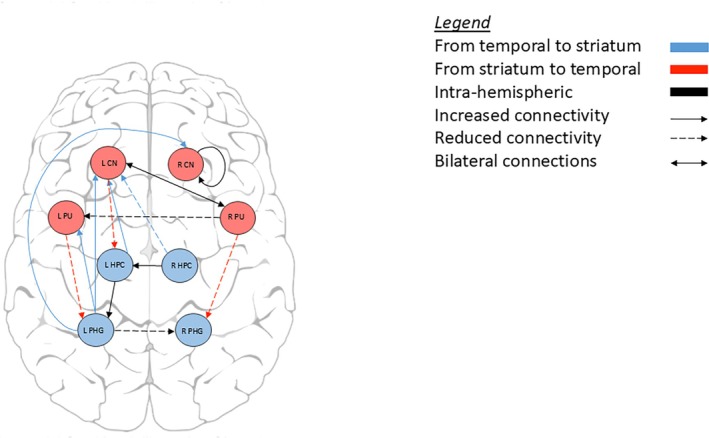
Effective connectivity between manifest Huntington's disease participants compared to controls. [Color figure can be viewed at wileyonlinelibrary.com]

Compared to healthy controls, manifest HD participants showed lower connectivity from the right hippocampus to left caudate nucleus and from the right putamen to left parahippocampal gyrus. We found a similar connectivity pattern from the striatum to the hippocampal formation in the premanifest HD group when compared to healthy controls, with lower connectivity from the right caudate nucleus to left hippocampus and lower connectivity from the left caudate nucleus to right hippocampus. In contrast, there was stronger connectivity from the right parahippocampal gyrus to right caudate nucleus in premanifest HD compared to healthy controls. All connections were inhibitory, except the excitatory connection from left caudate nucleus to right hippocampus in the premanifest HD group (Table 2, Figs. 2 and 3).

**TABLE 2:** Group differences in striatal‐temporal lobe connectivity and associations with object location memory performance

Valence	Connection	pHD vs. controls (↑/↓)	mHD vs. controls (↑/↓)	Connectivity effect size in Hz [95% CI]	Association with object location in pHD^a^	Association with object location in mHD^a^
Inhibitory	PHG_right_ → CN_right_	↑		0.15 [0.08–0.22]	0.11 [0.07–0.14]	
Inhibitory	CN_right_ → HPC_left_	↓		0.10 [−0.16 to −0.04]	−0.08 [−0.12 to −0.03]	
Excitatory	CN_left_ → HPC_right_	↓		0.11 [−0.16 to −0.06]	−0.04 [−0.06 to −0.02]	
Inhibitory	CN_left_ → CN_left_	↑		0.10 [0.02–0.19]	−0.10 [−0.15, −0.05]	
Inhibitory	HPC_right_ → CN_left_		↓	0.17 [−0.25 to −0.09]		−0.07 [−0.11 to −0.04]
Inhibitory	PU_right_ → PHG_right_		↓	0.12 [−0.20 to −0.04]		−0.14 [−0.17 to −0.12]
Excitatory	HPC_right_ → HPC_left_		↑	0.17 [0.10–0.24]		−0.04 [−0.06 to −0.01]
Inhibitory	PHG_left_ → PHG_right_		↓	0.19 [−0.28 to −0.10]		0.04 [0.02–0.07]
Inhibitory	CN_right_ → CN_right_		↑	0.19 [0.08–0.31]		0.11 [0.09–0.14]
Excitatory	PU_right_ → PU_left_		↓	0.09 [−0.17 to −0.02]		−0.07 [−0.10 to −0.05]

Abbreviations: pHD, premanifest Huntington's disease; mHD, manifest Huntington's disease; ↑, increase in connectivity; ↓, decrease in connectivity; PHG, parahippocampal gyrus; HPC, hippocampus; CN, caudate nucleus; PU, putamen.

^a^ These numbers represent normalized β coefficients. Negative association suggests worse object location memory and positive association suggests better performance.

We also found significant intra‐striatal and intra‐hippocampal formation connections in both HD groups when compared to controls. In the manifest HD group, there was stronger inhibition in the right caudate nucleus and excitatory decrease in connectivity from the right to left putamen. In the hippocampal formation of the manifest HD group, there was greater excitatory connectivity from right to left hippocampus and weaker inhibitory connectivity from left to right parahippocampal gyrus. In the premanifest HD group, there was stronger inhibition in the left caudate nucleus, but no significant intra‐hippocampal formation connections when compared to healthy controls.

### Object Location Memory Performance and Associations to Striatal‐Hippocampal Formation Effective Connectivity

Consistent with previous findings,^1^, ^11^ object location memory performance significantly differed between groups (*F*_(2,63)_ = 7.67, *P* = 0.001; controlling for gender), with both premanifest and manifest HD participants recalling significantly fewer test objects than healthy controls (premanifest HD: 8.72 ± 4.16 vs. controls: 12.41 ± 4.30, *P* = 0.008; manifest HD: 7.69 ± 4.25 vs. controls: 12.41 ± 4.30, *P* = 0.006). Performance did not differ significantly between premanifest and manifest HD groups (*P* = 1.0).

**FIG. 4:**
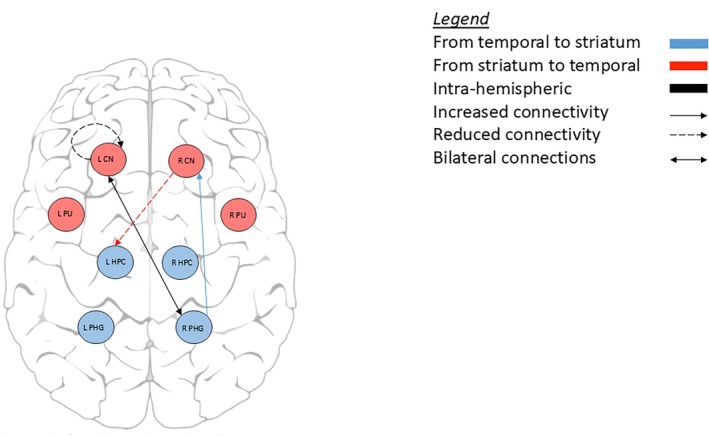
Effective connectivity associated with the object location memory task in premanifest Huntington's disease participants compared to controls. [Color figure can be viewed at wileyonlinelibrary.com]

**FIG. 5:**
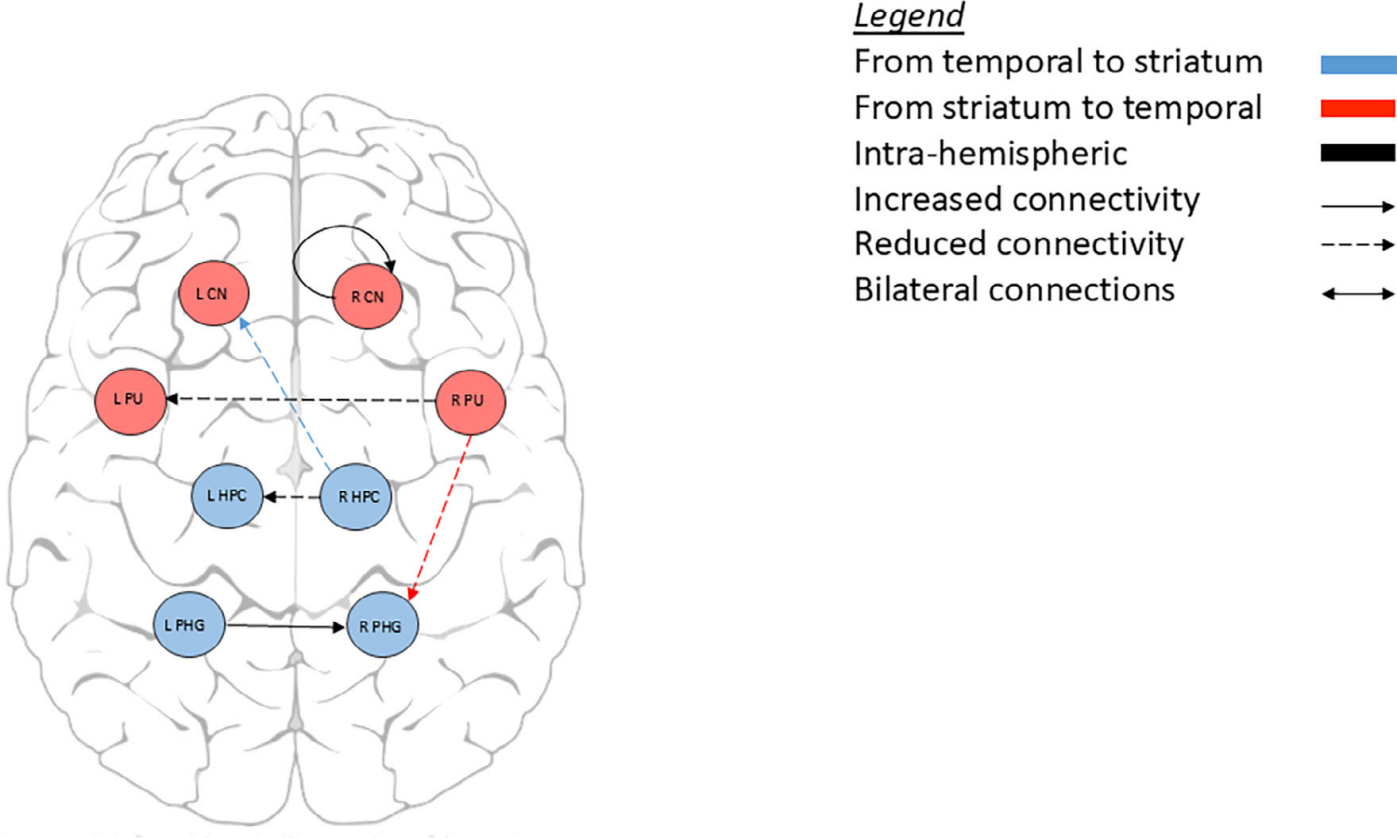
Effective connectivity associated with the object location memory task in manifest Huntington's disease participants compared to controls. [Color figure can be viewed at wileyonlinelibrary.com]

Object location memory in manifest HD was associated with lower connectivity from the right hippocampus to left caudate nucleus and from the right putamen to left parahippocampal gyrus. In those associations, worse memory performance was related to weaker striatal‐hippocampal formation connections. In premanifest HD, we found a similar pattern where worse memory was associated with weaker connectivity (ie, right caudate nucleus to left hippocampus; left caudate nucleus to right hippocampus). In contrast, hippocampal formation to striatum associations with object location memory showed that stronger connectivity from the right parahippocampal gyrus to right caudate nucleus was associated with better object location memory (Table 2, Figs. 4 and 5).

For intra‐striatal and intra‐hippocampal formation connectivity associations with object location memory, in manifest HD stronger connectivity within the right caudate nucleus was associated with better object location memory, and weaker right to left putamen connectivity was associated with worse performance. In the hippocampal formation of manifest HD, stronger connectivity from right to left hippocampus was associated with worse object location memory, and weaker connectivity from left to right parahippocampal gyrus was associated with better performance. In premanifest HD, increased connectivity within the left caudate nucleus was associated with worse object location memory performance.

## Discussion

Using spDCM, object location memory impairment in HD was associated with disruptions in effective connectivity between the striatum and hippocampal formation. Our findings do show that both premanifest and manifest HD participants exhibit reduced connectivity from the striatum to the hippocampal formation. However, there are key differences in directionality and lateralization. In premanifest HD, there is stronger connectivity from the parahippocampal gyrus to the striatum, which is associated with better object location memory, suggesting potential compensatory processes. Conversely, in manifest HD, the connectivity from the hippocampal formation to the striatum is reduced, and intra‐striatal and intra‐hippocampal connectivity patterns also show distinct alterations. These results suggest that although a general pattern of disrupted connectivity is present across HD stages, the specific direction and lateralization of these disruptions evolve as the disease progresses.

We found lower connectivity from the striatum to hippocampal formation in premanifest and manifest HD, which was associated with worse object location memory. This finding is not surprising given severe and early degeneration of the striatum in HD,^3^ however, we know of no previous HD research empirically demonstrating a link between the striatum and hippocampal formation and object location memory. Vice versa, connectivity from the hippocampal formation to striatum was lower in manifest HD, but in premanifest HD, connectivity from the parahippocampal gyrus to striatum was stronger and associated with increasing object location memory. The connectivity profile of the premanifest HD sample is commensurate with other task fMRI studies, which together with normal cognitive performance hypothesized neural compensation.^10^, ^35^, ^36^ The critical difference between our findings and previous studies is that premanifest HD participants performed significantly worse than healthy controls on the object location memory task while their effective connectivity was stronger, raising questions regarding the sensitivity of cognitive tasks for HD in the neural compensation hypothesis.

In HD, striatal volume loss is detectable up to 20 years before clinical diagnosis,^37^, ^38^, ^39^, ^40^, ^41^ yet cognitive decline becomes apparent approximately 10 years before diagnosis. Compensatory neural processes are thought to account for the preservation of cognitive function in the presence of brain pathology or differences in fMRI metabolic activity in task‐dependent regions when compared to controls.^16^, ^35^, ^42^, ^43^, ^44^, ^45^, ^46^ According to the neural compensation hypothesis, neurodegeneration and cognitive dysfunction are partially overcome by either reorganization of existing brain networks' connectivity (eg, increasing efficiency of information processing) and/or recruiting new regions to the network.^35^ The DCM literature is mixed with regard to compensation mechanism in premanifest HD. In the large multicenter TrackOn‐HD study,^47^ results were consistent with compensation and characterized by strengthened functional coupling between right dorsolateral prefrontal cortex and a left hemisphere network, which predicted better performance on the verbal n‐back task despite brain atrophy. In contrast, no indication of compensation was found for the motor network in a different premanifest HD cohort.^16^ Mixed compensation results are seen in a visuospatial working memory task with varying difficulty levels^48^ where premanifest HD made more errors at high difficulty level, but performed normally at low‐intermediate difficulty levels. In contrast to compensation, left intraparietal sulcus was reduced at high difficulty, with no fMRI over‐activation at lower task difficulty when compared to healthy controls. The changes we found in effective connectivity fits with neural atrophy in premanifest HD, suggesting the brain responds to pathology by altering how and where processing takes place.^35^ In contrast, our findings of object location memory impairment of premanifest HD does not fit the neural compensation pattern, suggesting the cognitive aspect of the neural compensation hypothesis should be revisited.

An alternative explanation to the increased effective connectivity in premanifest HD could be related to increased pathological connectivity such as glutamate excitotoxicity seen in premanifest HD. In the HD‐YAS (Huntington Disease — Young Adult Study) cohort,^49^ there was an increase in functional connectivity in the context of increasing cerebrospinal fluid neurofilament light (NfL), which is a marker of axonal degeneration that correlates with HD progression.^50^ McColgan et al^49^ also showed that regions with increased functional connectivity were also those with regional expression of genes specific to neuronal GABAergic and glutamatergic cells. This finding is consistent with evidence of neuronal network hyperexcitability driven by glutamatergic excitotoxicity and/or reduced inhibitory GABAergic activity in the earliest stages of neurodegeneration,^51^, ^52^ and suggesting that the increased effective connectivity that we have seen in our cohort may be pathological.

Cognitive changes in premanifest and early HD are subtle and selective for particular cognitive functions, and in later stages when neurodegeneration is extensive, a wide range of functions are affected. Early cognitive changes include psychomotor slowing, executive dysfunction, and inefficiencies in attention, working memory, and emotion recognition.^53^, ^54^, ^55^ Spatial memory impairment, and specifically, object location memory impairment, have only recently shown to be an early cognitive change^56^, ^57^ even in those who are far from disease onset and considered presymptomatic.^2^ Object location memory is the building block of environmental representation.^58^, ^59^ Successful recollection of object location requires several functional steps, including object processing, spatial location processing, and binding objects to locations.^60^ As such, object location memory is considered a special class of episodic memory, reflecting a form of contextual memory where object (identity) information is bound to location information.^60^ Deterioration of anterograde episodic memory is a well‐known early cognitive change in HD, which has been predominantly shown in the verbal domain.^55^ Given both the cognitive and connectivity findings, it may be that object location memory is more sensitive to detect cognitive changes in premanifest HD than previous tasks used in fMRI studies to demonstrate neural compensation.

Although DCM has not been used widely in HD neuroimaging studies, our results complement one previous cognitive DCM study showing altered effective connectivity in a large HD sample.^17^ Lahr et al^17^ showed decreased DCM connectivity in a network underlying working memory during a verbal n‐back fMRI with low‐high levels of working memory. The lowest levels of connectivity were at right dorsolateral prefrontal cortex in premanifest HD, and more so in early manifest HD. HD participants performed less accurately and more slowly at high working memory load compared with controls. Together, we suggest that cognitive impairment in HD may be related to connectopathy, abnormal interactions between brain regions mediating cognitive function and the pathophysiology of HD, in contrast to localized dysfunction within specific brain regions.^61^, ^62^ The ventral striatum receives direct projections from the hippocampus, and these striatum‐hippocampus interactions enable a flexible spatial response that combines egocentric and allocentric spatial processing during context‐dependent spatial decision making.^9^ HD neurodegeneration disrupts these striatal‐hippocampal connections, in turn impairing processing of spatial elements.

Neurocognitively, a limitation of our study is the focus on the striatum and hippocampal formation despite object location memory being mediated by a larger brain network. Our rationale for this focus was twofold^1^: there is an overlap in the involvement of these brain structures in object location memory and in HD, especially the striatum that is a central component in the spatial memory network^7^ and striatal degeneration is a hallmark HD neuropathology.^2^, ^63^ DCM is ROI‐based, hypothesis‐driven, inference technique where a few regions are chosen to limit the computational complexity of the model fitting and ensuring robust statistical inference especially for small sample sizes. Although our DCM and behavioral results were significant and consistent with the cognitive DCM study by Lahr et al,^17^ results are based on a smaller sample and should be interpreted with caution. Particular caution should be taken when interpreting results of the manifest HD group given the sample size of 12 participants.

We illuminate the neural correlates of a poorly characterized cognitive phenotype of HD, object location memory. The profile of effective connectivity linked to object location memory impairment in premanifest and manifest HD provides support to include object location memory in neuropsychological examination or clinical trials with cognitive endpoints, although more work is needed to show longitudinal change over the time period of a typical clinical trial. Object location memory is used in preclinical cognitive assessments in rodents, which precede clinical trials in humans, to assess the effects of potential treatments.^64^, ^65^, ^66^ The availability of a cognitive construct sensitive in both HD animal models and humans can align better cognitive testing across species in HD clinical trials and increase the likelihood that what is observed in the mouse will be seen in people.

## Author Roles

(1) Research project: A. Conception, B. Organization, C. Execution; (2) Statistical Analysis: A. Design, B. Execution, C. Review and Critique; (3) Manuscript Preparation: A. Writing of the First Draft, B. Review and Critique.

Y.G.J: 1A, 1B, 1C, 2A, 2B, 2C, 3A

G.D: 1C, 2B, 2C, 3A

T.B: 2B, 2C, 3B

J.C.S: 3B

A.R: 1A, 1B, 1C, 2A, 2B, 2C, 3B

## Financial Disclosures

Y.G.J. has no financial disclosures. G.D. has no financial disclosures. T.B. was funded by The Australian Government Research Training Program scholarship. J.C.S. has provided paid consulting services through Stout Neuropsych Pty Ltd to the following pharmaceutical sponsors in the past 12 months, including Skyhawk, Therapeutics, uniQure NV, LifeEdit Therapeutics, and Sage Therapeutics. J.C.S. is also a Director of Zindametrix Pty Ltd, a contract research organisation that provides services for implementation of cognitive assessments in HD clinical trials and has received compensation for Zindametrix activities in the past 12 months. A.R. is funded by the Australian Research Council (DE170100128 and DP200100757) and National Health and Medical Research Council (1194910) and CIFAR, Brain, Mind, and Consciousness Program.

## Supporting information


**Data S1.** Supporting Information.

## Data Availability

The data that support the findings of this study are available on request from the corresponding author. The data are not publicly available due to privacy or ethical restrictions.
