# Supplementary material for: Neural Mechanisms of Object Location Memory in Huntington's Disease
Source: Mov Disord. 2025 May 28;40(8):1692–703. doi: 10.1002/mds.30232 (PMC12371679; doi:10.1002/mds.30232)
Supplement: Supplementary file 1 — Data S1. Supporting Information. [file MDS-40-1692-s001.docx]

**Spectral Dynamic Causal Modelling (DCM)**

We characterised each subject’s distributed neuronal dynamics using spectral dynamic causal modelling (sDCM; Friston, Kahan, Biswal & Razi, 2014). DCM rests on a generative model of how interacting neural populations cause fMRI time series, which in turn give rise to functional connectivity measures (i.e., correlated hemodynamic fluctuations). DCM provides estimates of parameters quantifying the strength of directed connections between regions. We estimated these parameters at the between condition level using a Bayesian model (parametric empirical Bayes, PEB; Friston et al., 2016), enabling us to test for diagnostic (i.e., controls, HD, pre-HD) effects. Compared with functional connectivity approaches, which provide measures of correlation that cannot be used to infer causality, spectral DCM quantifies (directed) effective connectivity among brain regions.

**First level sDCM analyses**

This analysis provides measures of causal interactions between regions, as well as the amplitude and exponent of endogenous neural fluctuations within each region (Razi et al., 2015). Model inversion was based on standard variational Laplace procedures (Friston et al., 2007). This Bayesian inference method uses Free Energy as a proxy for (log) model evidence, while optimising the posterior density under Laplace approximation.

**Second Level sDCM analyses**

To characterise how group differences in neural circuitry were modulated by BMI and energy state, hierarchical models over the parameters were specified within a hierarchical Parametric Empirical Bayes (PEB) framework for DCM (Friston et al., 2016). all regressors and covariates were mean-centered so that the intercept of each model was interpretable as the mean connectivity.

Bayesian model reduction was used to test all combinations of parameters (i.e., reduced models) within each parent PEB model (assuming that a different combination of connections could exist [Friston et al., 2016]) and ‘pruning’ redundant model parameters. Parameters of the set of best-fit pruned models (in the last Occam’s window) were averaged and weighted by their evidence (i.e., Bayesian Model Averaging) to generate final estimates of connection parameters. To identify important effects (i.e., changes in directed connectivity), we compared models, using log Bayesian model evidence to ensure the optimal balance between model complexity and accuracy, with and without each effect and calculated the posterior probability for each model as a softmax function of the log Bayes factor. We treat effects (i.e., connection strengths and their changes) with a strong posterior probability > 0.99 (equivalent of very strong evidence in classical inference) as significant for reporting purposes.

Finally, in order to determine the predictive validity (e.g. whether BMI can be predicted from the final, reduced spDCM’s individual connections), leave-one-out cross validation was performed within the PEB framework (Zeidman et al., 2019). This procedure fits the PEB model in all but one participant and predicts the covariate of interest (e.g., homeostatic state) for the left-out participant. This is repeated with each participant to assess the averaged prediction accuracy for each model.

**References**

Friston, K. J., Kahan, J., Biswal, B., & Razi, A. (2014). A DCM for resting state fMRI.

Neuroimage, 94, 396-407.

Friston, K. J., Litvak, V., Oswal, A., Razi, A., Stephan, K. E., Van Wijk, B. C., ... &

Zeidman, P. (2016). Bayesian model reduction and empirical Bayes for group (DCM) studies. Neuroimage, 128, 413-431.

Friston, K., Mattout, J., Trujillo-Barreto, N., Ashburner, J., & Penny, W. (2007). Variational

free energy and the Laplace approximation. NeuroImage, 34(1), 220–234.

https://doi.org/10.1016/j.neuroimage.2006.08.035

Razi, A., Kahan, J., Rees, G., & Friston, K. J. (2015). Construct validation of a DCM for

resting state fMRI. NeuroImage, 106, 1–14. PubMed.

https://doi.org/10.1016/j.neuroimage.2014.11.027

Zeidman, P., Jafarian, A., Seghier, M. L., Litvak, V., Cagnan, H., Price, C. J., & Friston, K.

(2019). A guide to group effective connectivity analysis, part 2: Second level analysis

with PEB. NeuroImage, 200, 12–25
